# Anti-Müllerian hormone serum level and other markers associated with pregnancy outcome in oocyte donation

**DOI:** 10.1186/s12958-016-0138-0

**Published:** 2016-01-19

**Authors:** Anne-Sophie Delesalle, Geoffroy Robin, Patricia Thomas-Desrousseaux, Didier Dewailly, Sophie Catteau-Jonard

**Affiliations:** Departments of Endocrine Gynaecology and Reproductive Medicine, Hôpital Jeanne de Flandre, C.H.R.U., Faculty of Medicine, Université de Lille II, F-59000 Lille, France; INSERM U1172 Team 2, JPARC, Université de Lille II, F-59000 Lille, France

**Keywords:** Anti-Müllerian hormone, Oocyte donation, Donors, Pregnancy outcome, Antral follicle count

## Abstract

**Background:**

Oocyte donation is a medical technique used principally for woman with ovarian failure. Optimizing donor recruitment is essential to obtain the best results with this technique. Understanding how donor parameters influence outcome for the recipients is fundamental. The aim of this study was to determine whether clinical and/or biological parameters in the donors influence the chance of pregnancy in recipients. Our objective was also to verify whether the outcomes of controlled ovarian stimulation (COS) are predictive of pregnancy in the recipients.

**Methods:**

A retrospective observational study was conducted in the Department of Reproductive Medicine in the Lille University Hospital. Between September 2005 and April 2014, COS was performed in 145 donors for 308 recipients’ cycles. We compared the cycles whose outcome was pregnancy to the cycles without pregnancy. Quantitative variables were compared using the nonparametric Mann–Whitney test. Qualitative variables were compared using a Chi-2 test or Fisher exact test, according to the numbers. Covariance analysis was performed to adjust for potential confounding factors.

**Results:**

The donors who produced at least one pregnancy had a mean baseline serum anti-Müllerian hormone (AMH) level significantly higher than those who did not (*p* = 0.001). The mean antral follicle count did not differ between the 2 groups. After covariance analysis controlling for the number of couples attributed to a given donor, this difference remained significant (*p* = 0.029). Mature follicle number, estradiol serum level at the trigger day, number of mature oocytes and embryo number were significantly higher in the donors who produced pregnancy.

**Conclusion:**

Serum AMH level is associated with pregnancy outcome after oocyte donation.

## Background

Oocyte donation is a medical technique used principally for women with ovarian failure. At the end of 2011 in France, 1,806 couples were waiting for oocyte donation, whereas only 402 couples received a donation the same year. Recently, this activity increased slightly but insufficiently to address all requests, resulting in a long delay for recipient couples. Optimizing donor recruitment is essential to obtain the best results with this technique. Understanding how donor characteristics influence outcome of the recipients is fundamental. Many clinical or biological parameters can influence ART results such as the woman’s age, ovarian reserve and oocyte quality. Anti-Müllerian hormone (AMH) is an interesting biological marker because it reflects ovarian reserve. It can also predict ovarian response to controlled ovarian stimulation (COS). However the value of this biological marker to predict pregnancy in oocyte donation is unknown.

The aim of this study was to determine whether clinical and/or biological parameters in the donor influence the chance of pregnancy in recipients. We also verified whether the COS outcomes are predictive of pregnancy in the recipients. For this, we compared cycles resulting in pregnancy with cycles which did not result in pregnancy.

## Methods

All oocyte donation cycles performed in the Department of Reproductive Medicine in the Lille University Hospital between September 2005 and April 2014 were analyzed retrospectively. Oocyte donation cycles were performed in accordance with the bioethics law. This study was approved by the institutional Review Board of Lille University Hospital. All patients gave their informed consent before oocyte donation.

### Population

All potential oocyte donors underwent consultation with a referring physician of the Department. They were under the age of 38 years and had at least one child. About 25 % of the donors were altruistic. The other donors were relatives (i.e., recruited by recipient couple) but we performed anonymous cross-donations.

Donors with hereditary pathology, abnormal karyotype, body mass index (BMI) > 35 kg/m2, AMH < 1.5 ng/mL, AFC < 10 and/or abnormal serology were rejected. The donors underwent a rigorous clinical evaluation in search of contraindication to oocyte donation, including hereditary pathology. On the day of the consultation, ovarian reserve was evaluated by serum AMH assay (complemented by serum FSH and estradiol assays between days 2 and 5 of a spontaneous cycle), and antral follicle count (AFC, sum of both ovaries) by transvaginal ultrasonography (Voluson E8 Expert, GE Healthcare). Karyotype, psychological evaluation and HIV1-2, HTLV, HBV, HCV, syphilis, and CMV serology completed the evaluation.

Serum AMH levels were assessed using the second generation enzyme immunoassay AMH-EIA (ref. A16507) provided by Beckman Coulter Immunotech (Villepinte, France).

For matching donors and recipients, phenotypic characteristics (geographic origin, color of skin, eyes and hair, weight, height) and blood group were registered. A donor was allocated to one, two or three recipient(s) according to her ovarian reserve and the number of oocytes at the puncture.

### Donor cycle

All donors COS was performed in the department of Reproductive Medicine in University Hospital. One cycle was performed by one donor for one, two or three recipient’s couples.

The recipients received endometrial preparation synchronously to the donor stimulation using hormonal replacement treatment, and GnRH agonists if the recipient still cycled. Recipients were started on oral micronized estradiol (6 mg /day). Evaluation of endometrial thickness was performed at D14 and considered satisfactory if ≥ 7 mm. Vaginal micronized progesterone (800 mg/day) was initiated on the evening of donor oocyte retrieval.

A long agonist protocol was used for donors’ stimulation between September 2005 and December 2011. From January 2012, an antagonist protocol was used. COS was performed with gonadotropins, individually adjusted based on AFC, baseline serum AMH level, age and body mass index (BMI) values, and adapted during stimulation according to ultrasound findings and estradiol levels. When there were at least 2 follicles measuring > 18 mm mean diameter, ovulation was triggered either by subcutaneous recombinant hCG 0.25 mg (Ovitrel*; Merck Serono) or by GnRH agonist (triptoreline) 0.2 mg (Decapeptyl; Ipsen Pharma) in case of antagonist protocol. Follicles were aspirated transvaginally with ultrasound guidance 35 to 36 h thereafter. Fertilization was performed by intracytoplasmic sperm injection (ICSI) with the sperm of the spouse. Embryo transfer of 1 or 2 embryos was performed at days 2 or 3. If the embryo quality was sufficient, surplus embryos were frozen. The transfer of frozen embryos was performed under hormonal replacement protocol (oral estrogen and intravaginal progesterone) identical to the fresh transfer. Fourteen days after the embryo transfer, serum β-HCG level was measured. Transvaginal ultrasonography was performed at 5–6 weeks gestation to assess the presence of a gestational sac.

### Data collection

Data on age, BMI, parity, smoking, serum AMH levels and AFC were collected.

For each donor cycle, total dose of gonadotropins, duration of stimulation, number of follicles > 15 mm and estradiol serum level the day of trigger and the number of collected oocytes were recorded.

Clinical pregnancy was defined by the presence of an intrauterine gestational sac with heart activity on ultrasound performed at 5–6 weeks of amenorrhea.

Birth was defined as delivery of a child after at least 24 weeks of amenorrhea.

### Statistical analysis

Statistical analyses were performed using the Statistical Package for Social Sciences (SPSS 22.0 for Windows). Quantitative variables were compared using the nonparametric Mann–Whitney test. Qualitative variables were compared using a Chi-2 test or Fisher exact test, according to the numbers. Covariance analysis was performed to adjust for potential confounding factors.

## Results

Between September 2005 and April 2014, 145 donors underwent COS in our department for 308 recipients. One donor had COS cancelled because of ovarian hyperstimulation syndrome and 2 because of absence of ovarian response. For seven donors, we performed no embryo transfer due to poor oocyte or embryo quality. Over a period of 8 years, 418 transfer cycles were performed: 288 with fresh embryos (69 %) and 130 with frozen embryos (31 %).

Clinical, biological and ultrasound characteristics of the donors are summarized in Table 1. 28 % of donors were overweight or obese (BMI = 25-35 kg/m^2^) and 38 % were smokers.

**Table 1:** Clinical, biological and ultrasound characteristics of oocyte donors

Age (years)^a^	32 (23–37)^a^
Child number^a^	2 (1–4)^a^
BMI (kg/m^2^)^a^	23 (I8,5–31)^a^
AMH (ng/mL)^a^	3.9 (1,8–9.7)^a^
Antral follicle court (sum of both ovaries)^a^	20 (10–42.5]^a^
Smoker^b^	28 %
Overweight {BMI≥25kg/m^2^)^b^	38 %

^a^Median [5th percentile-55th percentile]

^b^Percent

The outcomes of the 145 donors COS are shown in Table 2. The donors whose eggs resulted in at least one pregnancy (n = 85) had serum AMH level significantly higher than those who did not (n = 60) (*p* = 0.001) (Table 3). After covariance analysis controlling for the number of couples attributed to a donor, the difference remained significant (*p* = 0.029). However, receiver operating characteristic (ROC) curve analysis showed that for pregnancy outcome, donors’ AMH values yielded a low area under the curve (0.665) [CI: 057–0.76], insufficient to reach a predictive threshold. For example, a threshold at 5 ng/mL gave a specificity of 75 % but a sensitivity of only 41 %. Concerning the stimulation parameters, mean mature follicle number, serum estradiol level at the trigger day, number of mature oocytes and embryo number were significantly higher in the donors who achieved pregnancy. The mean total dose of used gonadotropins was higher in donors without pregnancy. Conversely, stimulation duration did not differ significantly between the two groups (Table 3).

**Table 2:** Outcomes of the of the 145 donor’s COS

	Mean (SD)
Number of mature follicles	14.5 (±7)
Duration of stimulation (days)	12.2 (±1.7)
Estradiol on the trigger day (pg/mL)	2128 (±1142)
Total dose of gonadotropins (Ul)	2926 (±955)
Number of mature oocytes	9.6 (±6)
Number of embryos	6 (±4.1)
Pregnancies rate per donor	90 %
Birth rate per donor	70 %

**Table 3:** Comparisons between the donors achieving pregnancy and the others

	Pregnancy	No pregnancy	*P*
Number of oocyte donors	85	60	
Age (years)^a^	31 [23–37]	32 [24.9–37]	0.425
BMI ≥ 25	27.1 %	45.7 %	0.064
Smokers	37.3 %	37.5 %	0,897
Number of recipients per donor^a^	3 [1–3]	2 [1–4]	0,001
Child number of the donor^a^	2 [1–4]	1.9 [1–3.55]	0.096
AMH (ng/mL)^a^	4,48 [2.23–9.96]	3.15 [1.65–8.6]	0,001
Antral follicle count (sum of both ovaries)	20 [11–49]	19 [10–36]	0.095
Number of mature follicles^b^	16.6 ± 7.4	11.5 ± 5.2	<0.001
Duration of stimulation (days)^b^	12.1 ± 1.7	12.4 ± 1,8	0.26
Estradiol on the trigger day (pg/mL)^b^	2429 ± 1136	1694 ± 10.1	<0.001
Total dose of gonadotropins (UI)^b^	2650 ± 856	3315 ± 959	<0.001
Number of mature oocytes^b^	11.3 ± 6.1	7.2 ± 4.9	<0.001
Number of embryos^b^	7.5 ± 4.2	4 ± 3.2	<0.001

^a^Median [5th percentile–95th percentile]

^b^Mean ± standard deviation

The results of recipient cycles are summarized in Table 4. After the 145 donors’ COS, 1,380 mature oocytes were given to 308 recipients (mean ± SD: 4.5 ± 1.7 oocytes per recipient and per cycle). After fertilization by ICSI, 870 embryos were obtained, i.e. 2.9 ± 1.7 embryos per recipient. Among the embryos, 77 % were transferred. Among these, 73 % were transferred immediately and 27 % after thawing. A mean of 2.3 embryos were transferred to recipient per donor COS (immediately and after thawing), with an average of 1.6 embryos per transfer.

**Table 4:** Results of recipient cycles

	Value
Number of recipient cycles	308
Number of transfers	418
Number of pregnancies	133
Number of miscarriages	42
Number of births	108
Number of oocytes per recipient per cycle^a^	4,48 ± 1.68
Number of transferred embryos per cycle^a^	1.59 ± 0.51
Implantation rate	23 %
Pregnancy rate per transfer	31.8 %
Pregnancy rate per cycle (fresh and thawed embryos)	43.2 %
Birth rate per transfer	25.1 %
Miscarriage rate	31.6 %
Birth rate per cycle (fresh and thawed embryos)	35.1 %

^a^Mean ± standard deviation

## Discussion

The aim of this study was to identify whether features in the oocyte donors were associated with pregnancy in the recipient.

We did not observe an influence of donor’s age on pregnancy outcome, probably because most of the donors were young. Indeed, only 13.1 % of our donors were over 35 years old. In the literature, most of studies did not observe influence of donor’s age on oocyte donation outcome [1–4]. The use of ovarian reserve markers, such as serum AMH, to select the donors with a good potential ovarian response lead to refusal of older donors who more frequently have an AMH serum level lower than 1.5 ng/mL. Therefore, the age effect on pregnancy outcome is less important than in the setting of IVF in infertile women using their own oocytes.

We found a trend for a negative influence of donor’s BMI on pregnancy outcome. Overweight and obesity are responsible for impaired oocyte quality [5]. However, our results were not statistically significant and published data are controversial. We excluded donors having a BMI higher than 35 kg/m^2^, because of the difficulties and the risks linked to COS and puncture.

The high predictive value of serum AMH level on response to COS has already been reported [6, 7]. Our study was in agreement with other series of donors showing a good correlation between serum AMH level and ovarian response: number of collected oocytes, total dose of gonadotropins used and serum estradiol level the day of trigger [8–14]. Thus, serum AMH level is a reliable marker to obtain the largest number of oocytes while trying to limit the occurrence of ovarian hyperstimulation syndrome [8–12]. The minimum threshold of AMH at 1.5 ng/mL, that we used in our center, has been reported to provide a sensitivity of 86 % and a specificity of 78 % to predict a sufficient ovarian response [8].

In our study, the AFC was not different between the donors who achieved a pregnancy and those who did not, although serum AMH level was higher in the former. To our knowledge, this is the first study reporting a significantly higher AMH level in donors that produced a pregnancy compared with those who did not. Moreover, this difference persisted after adjustment for the number of recipients assigned to each donor, which would be a bias via the increased number of transfers. Previously, several authors have shown that serum AMH level was associated with oocyte quality, that could explain the association of AMH with pregnancy occurrence [15–19]. A recent meta-analysis reported a strong association between serum AMH level and the occurrence of live birth after in vitro fertilization, but the predictive value of AMH was very low [20]. Similarly in our study, we were not able to identify an AMH threshold value that could predict with sufficient power the outcome of oocyte donation. Indeed, the area under the ROC curve was low (AUC = 0.665; IC: 0.57-0.76). This is confirmed by five other studies [8, 9, 11, 13, 14]. For example, Riggs et al. found an AUC at 0.559 [8]. Thus, serum AMH level is associated with the outcome of an oocyte donation, but its predictive value is poor.

## Conclusion

Our study updates the importance of AMH to evaluate ovarian reserve of oocyte donors. AMH was shown to be correlated to the donor’s response to COS and to the number of oocytes allocated to one or more recipients. Although serum AMH level is associated with pregnancy outcome after oocyte donation, its predictive value is insufficient to be used to select donors by itself.

## Abbreviations

AFC: antral follicle count
AMH: anti-Müllerian hormone
ART: assisted reproductive technology
BMI: body mass index
COS: controlled ovarian hyperstimulation
ICSI: intracytoplasmic sperm injection
ROC: receiver operating characteristic
